# Dietary and physical activity adaptations to alternate day modified fasting: implications for optimal weight loss

**DOI:** 10.1186/1475-2891-9-35

**Published:** 2010-09-03

**Authors:** Monica C Klempel, Surabhi Bhutani, Marian Fitzgibbon, Sally Freels, Krista A Varady

**Affiliations:** 1 Department of Kinesiology and Nutrition, University of Illinois at Chicago, Chicago, IL, USA; 2 Department of Medicine, University of Illinois at Chicago, Chicago, IL, USA; 3 Department of Biostatistics, University of Illinois at Chicago, Chicago, IL, USA

## Abstract

**Background:**

Alternate day modified fasting (ADMF) is an effective strategy for weight loss in obese adults.

**Objective:**

The objective of this study was to examine the dietary and physical activity adaptations that occur during short-term ADMF, and to determine how these modulations affect rate of weight loss.

**Methods:**

Sixteen obese subjects (12 women/4 men) completed a 10-week trial consisting of 3 phases: 1) 2-week control phase, 2) 4-week ADMF controlled feeding phase, and 3) 4-week ADMF self-selected feeding phase.

**Results:**

Body weight decreased (*P *< 0.001) by 5.6 ± 1.0 kg post-treatment. Energy intake on the fast day was 26 ± 3% of baseline needs (501 ± 28 kcal/d). No hyperphagic response occurred on the feed day (95 ± 6% of baseline needs consumed, 1801 ± 226 kcal/d). Daily energy restriction (37 ± 7%) was correlated to rate of weight loss (*r *= 0.42, *P *= 0.01). Dietary fat intake decreased (36% to 33% of kcal, *P *< 0.05) with dietary counseling, and was related to rate of weight loss (*r *= 0.38, *P *= 0.03). Hunger on the fast day decreased (*P *< 0.05) by week 2, and remained low. Habitual physical activity was maintained throughout the study (fast day: 6416 ± 851 steps/d; feed day: 6569 ± 910 steps/d).

**Conclusion:**

These findings indicate that obese subjects quickly adapt to ADMF, and that changes in energy/macronutrient intake, hunger, and maintenance of physical activity play a role in influencing rate of weight loss by ADMF.

## Introduction

Rates of obesity have dramatically increased over the past three decades. At present, 34% of adults in the United States are obese (body mass index (BMI) > 30 kg/m^2^) [1]. According to the National Heart Blood and Lung Institute (NHLBI) Obesity Guidelines [2], dietary interventions should be implemented as the first line of treatment to help obese individuals lose weight. The most common diet therapy prescribed by practitioners is daily calorie restriction (CR). CR involves decreasing energy intake by 15 to 40% of baseline needs everyday. Evidence from short-term CR trials (8 to 24 weeks) demonstrate that CR is an effective means of decreasing body weight by 5 to 10% from baseline in obese patients [3-6].

Although CR is the most frequent diet strategy implemented to facilitate weight loss [7], many obese patients find it difficult to adhere to CR since food intake must be limited *every day*[8-10]. Alternate day modified fasting (ADMF) was created as an alternative to CR to improve compliance with dietary restriction regimens [11]. ADMF includes a "feed day" where food is consumed ad-libitum over a 24-h period, alternated with a "fast day", where food intake is partially reduced for 24-h. ADMF only requires an individual to restrict food intake on *every other day*, and as such, greatly increases adherence to these protocols. To date, four ADMF human trials have been performed [12-15], two of which were weight loss studies [13,14]. In the first trial by Johnson et al. [13], 8 weeks of modified ADMF, which allowed for 20% of energy needs to be consumed on the fast day, decreased body weight by 8% from baseline in overweight adults. In the second study conducted by our group [14], 8 weeks of modified ADMF (i.e. 25% energy intake on the fast day, alternated with an ad libitum feed day) resulted in a 6% weight loss in obese individuals. Although these preliminary findings suggest that ADMF may be an effective weight loss strategy, [13,14,16] what has yet to be examined is the dietary and physical activity adaptations that contributed to this pronounced weight loss by ADMF. Key questions that remain unresolved include: Are obese subjects able to dramatically change their meal pattern and limit their energy intake to 25% of needs on the fast day? If this is the case, what degree of hyperphagia occurs on the feed day in response to this lack of food on the fast day, and how does this affect net energy restriction and rate of weight loss? Moreover, how long does it take for obese subjects to become habituated to ADMF (i.e. no longer feel hungry on the fast day)? Furthermore, what changes in habitual physical activity occur during ADMF, and how do these changes affect rate of weight loss?

Accordingly, the objective of the present study was to examine the dietary and physical activity adaptations that occur during short-term ADMF, and to determine how these modulations affect rate of weight loss.

## Methods

### Subject selection

This study was approved by the Office for the Protection of Research Subjects at the University of Illinois, Chicago, and all volunteers gave their written informed consent prior to participation in the trial. As reported previously [14], participants were recruited by means of advertisements placed in community centers in the Chicago metropolitan area. Inclusion and exclusion criteria were assessed by an in-person interview. Participants meeting the following criteria were included in the study: age 35 to 65 y; body mass index between 30 and 39.9 kg/m^2^; weight stable for 3 months prior to the beginning of the study (i.e. less than 5 kg weight loss or weight gain); non-diabetic; no history of cardiovascular disease; lightly active (i.e. < 3 h/week of light intensity exercise at 2.5 to 4.0 metabolic equivalents (METs) for 3 months prior to the study); not participating in an exercise class; non-smoker; and not taking weight loss, lipid or glucose lowering medications. Peri-menopausal women were excluded from the study, and post-menopausal women (absence of menses for more than 2 y) were required to maintain their current hormone replacement therapy regimen for the duration of the study.

### Experimental design

Obese participants were enrolled in the study as a single cohort. Subjects participated in a 10-week trial consisting of three consecutive dietary intervention phases: (1) 2-week pre-loss control phase, (2) 4-week weight loss/ADMF controlled feeding phase, and (3) 4-week weight loss/ADMF self-selected feeding phase. During *Phase 1*, each subject maintained their usual eating and exercise habits in order to maintain a stable body weight. During *Phase 2*, subjects participated in a 4-week controlled-feeding ADMF period. All subjects consumed 25% of their baseline energy needs on the fast day (24 h), and then ate ad libitum on each alternating feed day (24 h). Individual baseline energy requirement was determined by the Mifflin equation [17]. Subjects were provided with a calorie-restricted meal on each fast day, and ate ad libitum at home on the feed day. The provided fast day meal was formulated for each subject using Nutritionist Pro Software (version 4.3, Axxya Systems, Stafford, TX). All diets were prepared in the metabolic kitchen at the Human Nutrition Research Unit (HNRU) at the University of Illinois, Chicago, and were provided as a 3-day rotating menu consisting of typical American foods. Meals provided on fast days during the controlled feeding phase are displayed in Table 1. Each feed/fast day began at midnight, and all fast day meals were consumed between 12.00 pm and 2.00 pm to ensure that each subject was undergoing the same duration of fasting. During *Phase 3*, all subjects participated in a self-selected feeding ADMF period in conjunction with weekly dietary counseling. This phase was put in place to determine if subjects could maintain the ADMF regimen on their own at home. During this phase, subjects still consumed 25% of their baseline energy needs on the fast day (between 12.00 pm and 2.00 pm), and ate ad libitum on the feed day. No food was provided to the subjects during this phase. Instead, a Registered Dietician met with each subject each week (for approximately 30 min per session) to develop individualized fast day meal plans. These plans included menus, portion sizes, and food lists that were consistent with each subject's food preferences and prescribed calorie levels for the fast day. Subjects were also instructed how to make healthy food choices on the ad libitum feed days, by choosing low fat meat and dairy options, and increasing fruit and vegetable intake.

**Table 1:** Meal components of provided fast day meals during controlled feeding phase

Foods	Fast day 1	Fast day 2	Fast day 3
Entree	Chicken fettuccini	Vegetarian pizza	Chicken enchilada
Fruit/vegetable	Carrot sticks	Apple	Orange
Snack	Cookie	Peanuts	Crackers

### Weight loss assessment

Body weight was measured weekly to the nearest 0.25 kg in the fasted state, without shoes, and in light clothing using a balance beam scale (HealthOMeter, Sunbeam Products, Boca Raton, FL).

### Reported food intake on feed days

Each participant completed a 3-day food record on 2 feed days during the week, and on 1 feed day during the weekend, at each week of the 10-week trial. Thus, a total of 30 feed day food records were collected for each subject. At baseline, the Research Dietician provided 15 min of instruction to each participant on how to complete the food records. These instructions included verbal information and detailed reference guides on how to estimate portion sizes and record food items in sufficient detail to obtain an accurate estimate of dietary intake. Subjects were instructed to record food items, in as much detail as possible, in the blank food diary provided. Any mixed foods were broken down to individual food items to be recorded one per line. Participants were not required to weigh foods but were asked to measure the volume of foods consumed with household measures (i.e. measuring cups and measuring spoons). When a commercial product was consumed, subjects were asked to indicate the weight of the product to assess portion size. Food records were collected at the weigh-in each week, and were reviewed by the Dietician for accuracy and completeness. All dietary information from the food records was entered into the food analysis program, Nutritionist Pro (Axxya Systems) by a single trained operator to alleviate inter-investigator bias. The program was used to calculate the total daily intake of energy, fat, protein, carbohydrate, cholesterol, and fiber.

### Reported food intake on fast days

During the ADMF controlled feeding phase, subjects were asked to report any additional food item consumed that was not included in the provided meal. During the ADMF self-selected feeding phase, each participant was asked to record his or her food intake on each fast day. At the beginning of this phase, the Research Dietician went over the food record instructions once again with each subject. Fast day food records were collected at the weigh-in each week, and all records were reviewed for accuracy and completeness by the Dietician. Dietary information from the fast day food records was analyzed by a single trained operator using Nutritionist Pro (Axxya Systems).

### Hunger, satisfaction with diet, and fullness assessment

Subjects completed a validated visual analog scale (VAS) on each fast day, in the evening, approximately 5 min before going to bed (reported bedtime ranged from 8.20 pm to 1.40 am) [18]. In brief, the VAS consisted of 100-mm lines, and subjects were asked to make a vertical mark across the line corresponding to their feelings from 0 (not at all) to 100 (extremely) for hunger, satisfaction with diet, or fullness. The VAS was collected at the weigh-in each week and reviewed for completeness. Quantification was performed by measuring the distance from the left end of the line to the vertical mark.

### Physical activity assessment

Habitual, free-living physical activity was assessed by a pedometer (Digiwalker SW-200, Yamax Corporation, Tokyo, Japan SW). Subjects wore the pedometer each day throughout the 10-week trial. The pedometer was worn attached to the participant's waistband during waking hours (except while bathing or swimming), and reset to zero each morning. Number of daily steps were recorded in a pedometer log provided, and the log was collected by study personnel at the weigh-in each week. No subjects were enrolled in an exercise class, and all participants were asked to refrain from joining any exercise programs during the course of the study. In this way, any changes in physical activity during the study could be estimated by the use of the pedometer.

### Statistics

Results are presented as means ± standard error of the mean (SEM). Tests for normality were included in the model. One-factor repeated measures analysis of variance was performed to determine an overall *P *value over time. The main variables tested included body weight, energy intake, nutrient intake, hunger, satisfaction and fullness. The Bonferroni correction was used to assess significance. Relations between continuous variables (i.e. body weight, energy intake, nutrient intake, hunger, satisfaction and fullness) were assessed by simple regression analyses as appropriate. Data were analyzed by using SPSS software (version 18.0 for Mac OS X; SPSS Inc., Chicago, IL).

## Results

### Subject characteristics at baseline

Of the 52 participants screened, 20 were deemed eligible to participate in the study, and 16 (4 men/12 women) completed the entire 10-week trial. Subjects who completed the study were middle age (46 ± 3 y, 35-65 y), obese (BMI 34 ± 1 kg/m^2^, 30.2-39.9 kg/m^2^), sedentary (2.4 ± 0.3 h/week of physical activity), and borderline hypercholesterolemic (LDL cholesterol level 106 ± 10 mg/dl). Eight participants were African-American, 2 were Caucasian, and 6 were Hispanic.

### Changes in body weight in response to ADMF

During the control phase, body weight remained stable (week 1: 96.4 ± 5.3 kg, week 2: 96.5 ± 5.2 kg). At the end of the ADMF controlled feeding phase (week 6), body weight decreased (*P *< 0.001) to 93.8 ± 5.0 kg (feed day measurement) and 93.7 ± 5.0 kg (fast day measurement). By the end of the ADMF self-selected feeding phase (week 10), body weight was further reduced (*P *< 0.001) to 92.8 ± 4.8 kg (feed day measurement) and 90.8 ± 5.0 kg (fast day measurement). Thus, a total weight loss of 5.6 ± 1.0 kg (-0.7 ± 1.0 kg per week) was attained after 8 weeks of ADMF.

### Degree of energy restriction achieved with ADMF and relation to body weight changes

**Figure 1:**
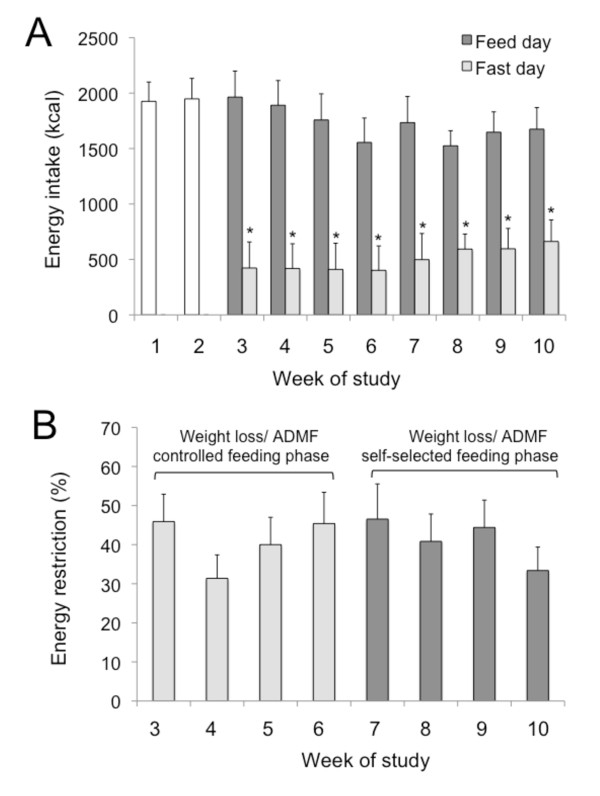
**Energy intake and energy restriction during each phase of the trial**. Values reported as mean ± SEM. **A**. Mean energy intake during the control phase, and on feed and fast days during each week of the trial. Energy intake did not change from the beginning to the end of the control phase. *P < 0.001 fast day energy intake was lower than feed day energy intake at each week (One-factor ANOVA with Bonferroni analysis). **B**. Mean percent energy restriction during each week of the study. No difference for percent energy restriction between weeks (One-factor ANOVA with Bonferroni analysis).

Energy intake and percent energy restriction were determined from food record data collected on feed and fast days. Mean completion rate of feed and fast day food records was 83 ± 5%, and 86 ± 4%, respectively. Energy intake on feed and fast days during each week of the trial is displayed in Figure 1A. During the control phase, mean energy intake was 1937 ± 180 kcal. Mean feed day energy intake (1801 ± 226 kcal) at each week of the trial was similar to that of the control phase, and did not differ between ADMF controlled-feeding and self-selected feeding phases. Mean energy intake on the fast day (501 ± 28 kcal, 26 ± 3% of baseline needs consumed) was lower (*P *< 0.001) than that of the feed day at each week of the trial. The ratio of energy consumed on the fast day versus the feed day during the controlled feeding phase (0.28 ± 0.03) did not differ from that of the self-selected feeding phase (0.30 ± 0.05). Percent energy restriction is reported in Figure 1B. Over the course of the trial, percent daily energy restriction remained high and stable (37 ± 7%), and did not differ between the ADMF controlled-feeding and self-selected feeding phases. Degree of energy restriction achieved by ADMF was correlated to rate of weight loss (*r *= 0.42, *P *= 0.01) and absolute post-treatment weight loss (*r *= 0.48, *P *= 0.008).

### Hyperphagic response

**Figure 2:**
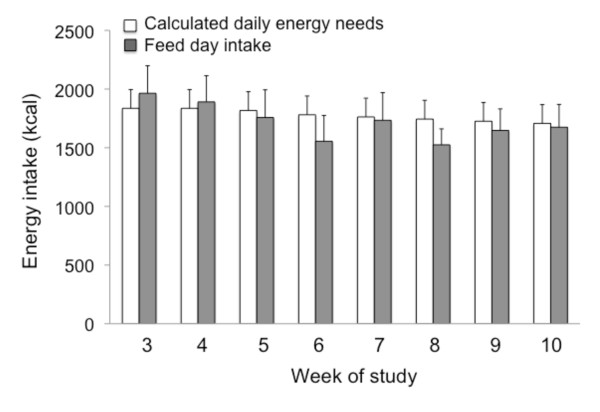
**Hyperphagic response on the feed day to lack of food on the fast day**. Values reported as mean ± SEM. Calculated daily energy needs assessed for each subject using the Mifflin equation. No difference between feed day energy intake and calculated energy requirement at any week of the trial (One-factor ANOVA with Bonferroni analysis).

Hyperphagia on the feed day in response to the lack of food on the fast day is reported in Figure 2. We hypothesized that the participants would increase their energy intake on the feed day by approximately 125% of their baseline needs. However, no such hyperphagic response was observed, as mean feed day energy intake (1801 ± 226 kcal) was similar to calculated requirements (1896 ± 160 kcal) at each week of the trial. Thus, on average, subjects were only consuming 95 ± 6% of their calculated energy needs on the feed day.

### Changes in nutrient intake during ADMF and relation to body weight changes

The nutrient composition of feed and fast day meals during each phase of the trial is displayed in Table 2. During the control phase, subjects were consuming a high fat (> 35% of kcal), high saturated fat (> 7% of kcal), high cholesterol (> 200 mg/d), and low fiber diet (< 25 g/d), as per the National Cholesterol Education Program (NCEP) dietary guidelines [19]. During the ADMF controlled feeding phase, the nutrient composition of feed day diet was similar to that of the control phase (i.e. high total fat, high saturated fat, high cholesterol and low fiber). During the ADMF self-selected feeding phase, total fat (33 ± 4% kcal) and saturated fat (7 ± 1% kcal) intake on the feed day decreased (*P *< 0.05), relative to the control phase. Dietary cholesterol, however, was still above the recommended daily allowance (223 ± 27 mg/d), and dietary fiber (15 ± 1 g) was still below the recommended intake level on the feed day. Decrease in total fat intake was related to rate of weight loss (*r *= 0.38, *P *= 0.03).

**Table 2:** Nutrient composition of feed day and fast day meals during each phase of the trial^1^

	Pre-loss control phase^2^	Weight loss/ADMF controlled feeding phase		Weight loss/ADMF self-selected feeding phase	
		**Feed day^2^**	**Fast day^3^**	**Feed day^2^**	**Fast day^2^**

Energy (kcal)	1937 ± 180	1792 ± 228	413 ± 20	1645 ± 187	588 ± 46
Protein (% kcal)	18 ± 1	18 ± 1	23 ± 1	19 ± 1	20 ± 1
Carbohydrate (% kcal)	46 ± 3	47 ± 3	52 ± 0	46 ± 2	51 ± 3
Total fat (% kcal)	36 ± 5^a^	36 ± 6^a^	25 ± 1^b^	33 ± 4^b^	29 ± 1^b^
Saturated fat (% kcal)	11 ± 1^a^	10 ± 1^a^	6 ± 1^b^	7 ± 1^b^	9 ± 1^a^
Monounsaturated fat (% kcal)	11 ± 1	12 ± 1	11 ± 1	13 ± 1	8 ± 1
Polyunsaturated fat (% kcal)	10 ± 2	11 ± 1	8 ± 1	10 ± 1	9 ± 1
Trans fat (% kcal)	4 ± 1^a^	3 ± 1^a^	0^b^	3 ± 1^a^	3 ± 1^a^
Cholesterol (mg)	249 ± 46^a^	239 ± 24^a^	68 ± 3^b^	223 ± 27^a^	73 ± 9^b^
Cholesterol (mg/kcal)	0.13 ± 0	0.13 ± 0	0.17 ± 0	0.14 ± 0	0.12 ± 0
Fiber (g)	16 ± 2^a^	12 ± 2^a^	10 ± 1^b^	15 ± 1^a^	7 ± 1^b^
Fiber (g/kcal)	0.008 ± 0	0.008 ± 0	0.02 ± 0	0.009 ± 0	0.01 ± 0

^1 ^Values reported as mean ± SEM. Values in the same row with different superscript letters are significantly different, *P *<0.05 (One-factor ANOVA with Bonferroni analysis).

^2 ^Food intake self-reported each week using 3-d food record.

^3 ^Food was provided on the fast day during the controlled feeding phase.

### Hunger, satisfaction with diet, and fullness

**Figure 3:**
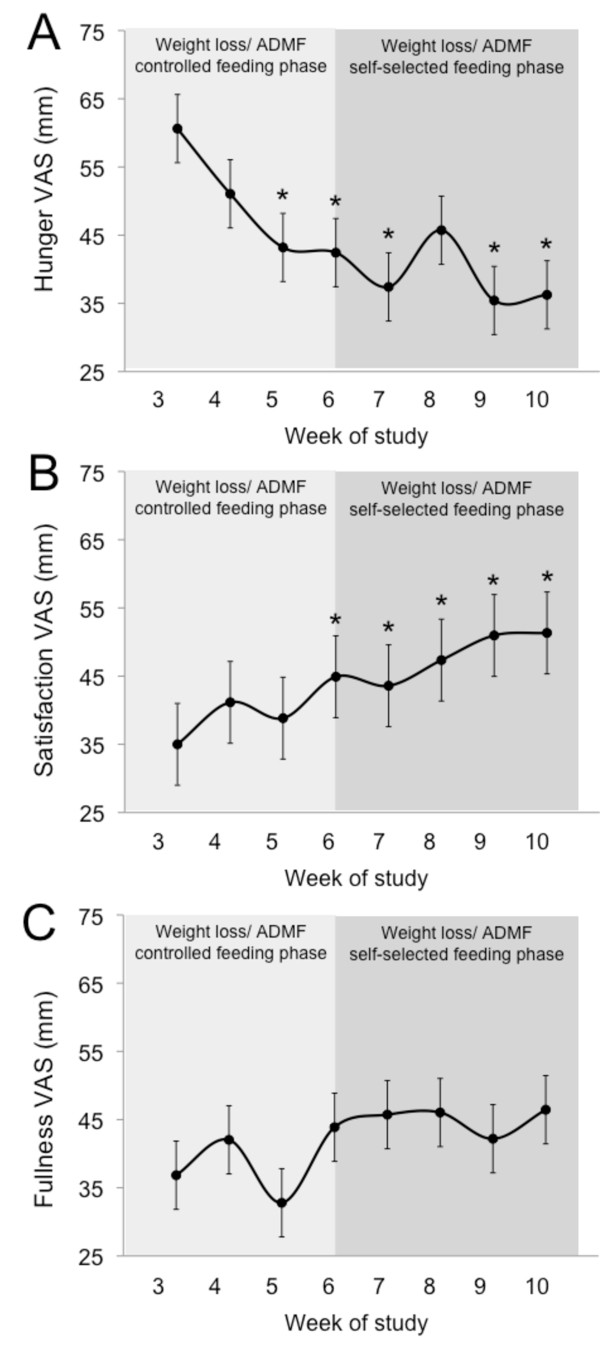
**Hunger, satiety and fullness during each phase of the trial**. Values reported as mean ± SEM. **A**. Hunger scores at each week of the study. **B**. Satisfaction with diet scores at each week of the study. **C**. Fullness scores at each week of the study. *P < 0.05, relative to week 3 (One-factor ANOVA with Bonferroni analysis).

Changes in hunger, satisfaction, and fullness during the trial are displayed in Figure 3. During the first week of ADMF, hunger scores were elevated. However, after two weeks of ADMF, hunger scores decreased (*P *< 0.05) and remained low throughout the rest of the trial. Satisfaction with the ADMF diet was low during the first 4 weeks of the intervention, but gradually increased (*P *< 0.05) during the last 4 weeks of the study. Fullness scores remained low during the entire 8-week ADMF intervention.

### Changes in physical activity habits

**Figure 4:**
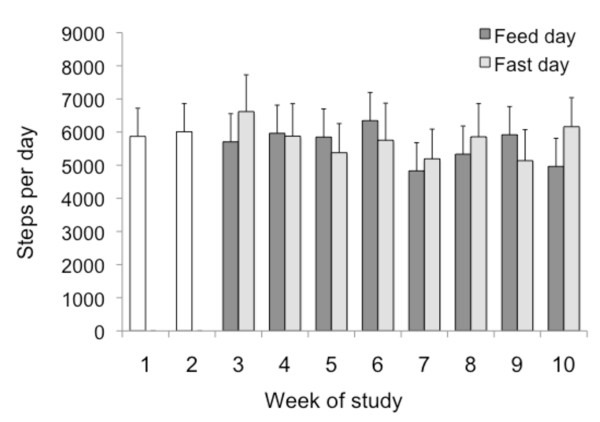
**Physical activity level of subjects at each week of the trial measured as steps per day**. Values reported as mean ± SEM. Steps/d recorded by a pedometer worn daily. No changes in number of steps/d taken over the course of the 10-week trial, and no difference between feed and fast day values (One-factor ANOVA with Bonferroni correction).

All subjects wore a pedometer each day throughout the entire trial to assess changes in physical activity habits. On average, subjects were very compliant with pedometer use, and steps were recorded on 87 ± 4% of study days. We hypothesized that subjects would feel less energetic on the fast days, and would therefore take less steps/d on fast days than feed days. Interestingly, no difference was noted when fast day values (6416 ± 851 steps/d) were compared to feed day values (6569 ± 910 steps/d) (Figure 4). Moreover, physical activity remained constant throughout the 10-week study, as steps/d taken during the control phase was similar to that of the ADMF phases.

## Discussion

Preliminary reports indicate that ADMF may be an effective strategy to help obese individuals lose weight [13,14]. However, the dietary and physical activity adaptations that contributed to this pronounced weight loss by ADMF were not tested previously. We show here, for the first time, that weight loss by ADMF occurred due to change in meal pattern, i.e. obese subjects limited their energy intake to 25% of needs on the fast day with no hyperphagic response on the feed day. This change in meal pattern helped these subjects to achieve a marked degree of energy restriction (37% net daily) which was related to the pronounced weight loss attained (5.6 kg in 8 weeks). This study is also the first to demonstrate that subjects become habituated to the ADMF diet (i.e. feel very little hunger on the fast day) after approximately 2 weeks, and that physical activity habits are not affected by fasting on alternate days.

A key objective of the present study was to examine the degree of energy restriction achieved by ADMF and to investigate how this relates to rate of weight loss. In order to measure energy intake and percent energy restriction, we asked obese participants to complete food records on feed and fast days throughout the trial. Results from the food record analysis reveal that obese subjects were able to consistently limit their energy intake to approximately 25% of needs (500 kcal) on the fast day. Our data also show that the ratio of energy consumed on the fast day versus the feed day did not differ between phases. However, it should be noted that there was a trend towards consuming less energy on the feed days, and more energy on the fast days over the course of the trial. It will therefore be of interest in long-term ADMF studies to examine whether restriction gradually diminishes on the fast day after several months of diet. The degree of hyperphagia that occurred on the feed day in response to the lack of food on the fast day was also assessed. Our data indicate that no hyperphagic response took place as subjects only consumed approximately 95% of their calculated energy needs on each feed day throughout the trial. These findings therefore suggest that obese subjects are able to drastically change their meal pattern in a way that conforms to the ADMF protocol. Nevertheless, there are several limitations to these data that must be discussed. First and foremost, it is well known that obese subjects underreport energy intake by 20 to 40% when completing food records [20,21]. The extent to which these subjects underreported energy intake became apparent when we tried to relate reported energy intake to the weight loss achieved. From the food record data, we calculated that, on average, subjects were restricted by 37% of calculated needs every day. If the subjects were indeed restricted by this amount, this would have resulted in a rate if weight loss of 1.2 kg/week. In actuality, the rate of weight loss was 0.7 kg/week. This disparity between reported intake and weight loss can be observed when examining the limited amount of weight lost during the 4-week self-selected feeding phase (body weight reduction of 93.8 kg to 92.8 on the feed day, equivalent to 1 kg of weight loss). The incongruity between self-reported energy intake and rate of weight loss therefore suggests that subjects were underreporting energy intake. In view of this, it will be important for future ADMF trials to assess energy intake and energy restriction by more robust methods, such as the doubly labeled water technique [22,23]. It should also be mentioned that assessing body weight changes by ADMF is difficult as weight measurements are drastically different from feed to fast day. This discrepancy in body weight is most likely due to the additional weight of food present in the gastrointestinal tract, and not changes in fat mass from day to day. As a potential solution, future trials of ADMF should average body weight measurements taken from consecutive feed and fast days to attain a more accurate assessment of weight.

In addition to energy intake, we also examined changes in dietary macronutrient composition throughout the course of the trial. We hypothesized that during the ADMF controlled feeding phase (weeks 3-6), when dietary counseling was not provided, subjects would instinctively choose higher fat/more energy dense foods on the feed day to make up for the lack of energy consumed on the fast day. Interestingly, fat intake did not increase from the baseline period (36% of kcal) to the ADMF-controlled feeding period (36% of kcal). These preliminary data suggest that subjects are not likely to consume higher fat diets on the feed day when partaking in an ADMF regimen. We also hypothesized that the dietary counseling provided during the self-selected feeding phase (weeks 7-10), would help subjects decrease total fat, saturated fat, and cholesterol intake, while increasing fiber intake. Results reveal that counseling assisted these individuals in lowering their total fat and saturated fat intakes to levels that conform with NCEP dietary recommendations [19], and that these changes in fat intake were related to rate of weight loss. On the other hand, dietary counseling appeared to have no effect on cholesterol or fiber intake. This lack of effect of dietary counseling on the intakes of these nutrients has been reported previously [24]. It should also be noted that fiber intake on the fast day was particularly low (7-10 g/d). Since quantity of food consumed on the fast day is limited, it would be difficult for individuals to meet fiber requirements [25]. As such, it is recommended that future trials in the ADMF field provide a fiber supplement on the fast day to help individuals meet recommendations [19,26].

Changes in perceived hunger, satisfaction with diet, and fullness were also evaluated on each fast day throughout the trial. This study is the first to show that obese subjects become habituated with ADMF after approximately 2 weeks of diet (i.e. feel very little hunger on the fast day). Our data also demonstrate that subjects become more satisfied with ADMF after approximately 4 weeks of diet. Feelings of fullness, however, remained low across the course of the trial suggesting that subjects never felt "full" at any point while undergoing 8-weeks of ADMF. These findings may have important implications for long-term adherence to ADMF by obese men and women [27-29]. More specifically, since hunger virtually diminishes, and since satisfaction with diet considerably increases within a short amount of time (2-4 weeks), it is likely that obese participants would be able to follow the diet for longer periods of time. It is important to note, however, that the subjects only completed the VAS scales pre-bedtime. Thus, the data only reflects their feelings immediately before going to bed, and is not indicative of their feelings of hunger and satisfaction throughout the day. Future trials in this area should administer these VAS scales throughout the day to obtain a more complete data set for these variables. It should also be noted that hunger spiked at week 8. We speculate that this may have occurred because this study week corresponded to Memorial Day weekend, and subjects may have felt hungrier while attending food-related celebrations. Moreover, trials examining the ability of obese subjects to comply with ADMF for longer durations (i.e. 24 to 52 weeks), and in consequence, lose larger amounts of weight, will be an important focus of future research in this field.

The effects of ADMF on habitual physical activity was also assessed by having the subjects wear a pedometer on everyday of the study. We hypothesized that subjects would feel less energetic on the fast days, and would therefore be less physically active (i.e. take less steps/d) on fast days than feed days. Surprisingly, physical activity level did not differ between feed and fast days. Moreover, there was no difference in activity level when steps/d taken during the ADMF phase were compared to steps/d taken during the control phase. Similar results have also been reported in normal weight individuals undergoing ADMF [12]. These data suggest that obese individuals are able to maintain their level of habitual physical activity despite decreases in energy intake on the fast day. This maintenance of physical activity while undergoing ADMF would thus allow obese individuals to lose weight consistently on feed and fast days as energy expenditure would stay constant.

A key limitation of this study is that there was no true control group. Having a control arm run parallel to the treatment (ADMF) arm would have strengthened the study by allowing us to: 1) compare changes in the ADMF group to that of a non-restricted control group at each time point, and 2) identify events (such as holidays) that may have resulted in deviations from the prescribed diet. Future studies aiming to test similar objectives should employ a control group where possible.

In summary, these findings indicate that obese subjects quickly adapt to ADMF, and that changes in energy/macronutrient intake, hunger level, and maintenance of physical activity play a role in influencing rate of weight loss by ADMF. These preliminarily data offer promise for the implementation of ADMF as a long-term weight loss strategy in obese populations.

## Competing interests

The authors declare that they have no competing interests.

## Authors' contributions

MCK performed all the diet analyses and assisted with trial coordination and manuscript preparation. SB coordinated the human clinical trial. MF and SF assisted with data analysis and manuscript preparation. KAV designed the study and wrote the manuscript. All authors read and approved the final manuscript.

## References

[B1] Overweight and Obesity Trends Among Adults 2009 Atlanta, GA: Centers for Disease Control and Prevention

[B2] Clinical Guidelines on the Identification, Evaluation, and Treatment of Overweight and Obesity in Adults: The Evidence Report 1998 NHLBI Publication #98-4083

[B3] Nicklas BJ Wang X You T Lyles MF Demons J Easter L Berry MJ Lenchik L Carr JJ Effect of exercise intensity on abdominal fat loss during calorie restriction in overweight and obese postmenopausal women: a randomized, controlled trial Am J Clin Nutr 2009 89 4 1043 1052 10.3945/ajcn.2008.26938 19211823 PMC2667455

[B4] Kirk E Reeds DN Finck BN Mayurranjan SM Patterson BW Klein S Dietary fat and carbohydrates differentially alter insulin sensitivity during caloric restriction Gastroenterology 2009 136 5 1552 1560 10.1053/j.gastro.2009.01.048 19208352 PMC2677125

[B5] Parra D Bandarra NM Kiely M Thorsdottir I Martinez JA Impact of fish intake on oxidative stress when included into a moderate energy-restricted program to treat obesity Eur J Nutr 2007 46 8 460 467 10.1007/s00394-007-0686-3 18026868

[B6] Ho JT Keogh JB Bornstein SR Ehrhart-Bornstein M Lewis JG Clifton PM Torpy DJ Moderate weight loss reduces renin and aldosterone but does not influence basal or stimulated pituitary-adrenal axis function Horm Metab Res 2007 39 9 694 699 10.1055/s-2007-985354 17846979

[B7] Steyer TE Ables A Complementary and alternative therapies for weight loss Prim Care 2009 36 2 395 406 19501250 10.1016/j.pop.2009.01.011

[B8] Dansinger ML Gleason JA Griffith JL Selker HP Schaefer EJ Comparison of the Atkins, Ornish, Weight Watchers, and Zone diets for weight loss and heart disease risk reduction: a randomized trial JAMA 2005 293 1 43 53 10.1001/jama.293.1.43 15632335

[B9] Das SK Gilhooly CH Golden JK Pittas AG Fuss PJ Cheatham RA Tyler S Tsay M McCrory MA Lichtenstein AH Long-term effects of 2 energy-restricted diets differing in glycemic load on dietary adherence, body composition, and metabolism in CALERIE: a 1-y randomized controlled trial Am J Clin Nutr 2007 85 4 1023 1030 17413101 10.1093/ajcn/85.4.1023

[B10] Fardet L Flahault A Kettaneh A Tiev KP Toledano C Lebbe C Cabane J [Systemic corticosteroid therapy: patients' adherence to dietary advice and relationship between food intake and corticosteroid-induced lipodystrophy] Rev Med Interne 2007 28 5 284 288 10.1016/j.revmed.2006.12.013 17391811

[B11] Varady KA Hellerstein MK Alternate-day fasting and chronic disease prevention: a review of human and animal trials Am J Clin Nutr 2007 86 1 7 13 17616757 10.1093/ajcn/86.1.7

[B12] Halberg N Henriksen M Soderhamn N Stallknecht B Ploug T Schjerling P Dela F Effect of intermittent fasting and refeeding on insulin action in healthy men J Appl Physiol 2005 99 6 2128 2136 10.1152/japplphysiol.00683.2005 16051710

[B13] Johnson JB Summer W Cutler RG Martin B Hyun DH Dixit VD Pearson M Nassar M Telljohann R Maudsley S Alternate day calorie restriction improves clinical findings and reduces markers of oxidative stress and inflammation in overweight adults with moderate asthma Free Radic Biol Med 2007 42 5 665 674 10.1016/j.freeradbiomed.2006.12.005 17291990 PMC1859864

[B14] Varady KA Bhutani S Church EC Klempel MC Short-term modified alternate day fasting: A novel dietary strategy for weight loss and cardio-protection in obese adults Am J Clin Nutr 2009 90 5 1138 1143 10.3945/ajcn.2009.28380 19793855

[B15] Heilbronn LK Smith SR Martin CK Anton SD Ravussin E Alternate-day fasting in nonobese subjects: effects on body weight, body composition, and energy metabolism Am J Clin Nutr 2005 81 1 69 73 15640462 10.1093/ajcn/81.1.69

[B16] Bhutani S Klempel MC Berger RA Varady KA Improvements in Coronary Heart Disease Risk Indicators by Alternate-Day Fasting Involve Adipose Tissue Modulations Obesity (Silver Spring) 2010 in press 20300080 10.1038/oby.2010.54

[B17] Mifflin MD St Jeor ST Hill LA Scott BJ Daugherty SA Koh YO A new predictive equation for resting energy expenditure in healthy individuals Am J Clin Nutr 1990 51 2 241 247 2305711 10.1093/ajcn/51.2.241

[B18] Flint A Raben A Blundell JE Astrup A Reproducibility, power and validity of visual analogue scales in assessment of appetite sensations in single test meal studies Int J Obes Relat Metab Disord 2000 24 1 38 48 10.1038/sj.ijo.0801083 10702749

[B19] Third Report of the Expert Panel on Detection, Evaluation, and Treatment of High Blood Cholesterol in Adults (ATP III Final Report) 2002 NHLBI Publication # 02-5215. National Institutes of Health 1 280

[B20] Kretsch MJ Fong AK Green MW Behavioral and body size correlates of energy intake underreporting by obese and normal-weight women J Am Diet Assoc 1999 99 3 300 306 quiz 307-308 10.1016/S0002-8223(99)00078-4 10076581

[B21] Goris AH Westerterp-Plantenga MS Westerterp KR Undereating and underrecording of habitual food intake in obese men: selective underreporting of fat intake Am J Clin Nutr 2000 71 1 130 134 10617957 10.1093/ajcn/71.1.130

[B22] Schoeller DA Recent advances from application of doubly labeled water to measurement of human energy expenditure J Nutr 1999 129 10 1765 1768 10498745 10.1093/jn/129.10.1765

[B23] Surrao J Sawaya AL Dallal GE Tsay R Roberts SB Use of food quotients in human doubly labeled water studies: comparable results obtained with 4 widely used food intake methods J Am Diet Assoc 1998 98 9 1015 1020 10.1016/S0002-8223(98)00232-6 9739802

[B24] Sacks FM Bray GA Carey VJ Smith SR Ryan DH Anton SD McManus K Champagne CM Bishop LM Laranjo N Comparison of weight-loss diets with different compositions of fat, protein, and carbohydrates N Engl J Med 2009 360 9 859 873 10.1056/NEJMoa0804748 19246357 PMC2763382

[B25] Hyman FN Sempos E Saltsman J Glinsmann WH Evidence for success of caloric restriction in weight loss and control. Summary of data from industry Ann Intern Med 1993 119 7 Pt 2 681 687 8363197 10.7326/0003-4819-119-7_part_2-199310011-00011

[B26] Dietary Reference Intakes for Energy, Carbohydrate, Fiber, Fat, Fatty Acids, Cholesterol, Protein, and Amino Acids 2005 National Academy of Sciences. Institute of Medicine. Food and Nutrition Board 10.1016/s0002-8223(02)90346-9 12449285

[B27] Anton SD Han H York E Martin CK Ravussin E Williamson DA Effect of calorie restriction on subjective ratings of appetite J Hum Nutr Diet 2009 22 2 141 147 10.1111/j.1365-277X.2008.00943.x 19302119 PMC2712828

[B28] Drapeau V King N Hetherington M Doucet E Blundell J Tremblay A Appetite sensations and satiety quotient: predictors of energy intake and weight loss Appetite 2007 48 2 159 166 10.1016/j.appet.2006.08.002 17045700

[B29] Drapeau V Blundell J Therrien F Lawton C Richard D Tremblay A Appetite sensations as a marker of overall intake Br J Nutr 2005 93 2 273 280 10.1079/BJN20041312 15788121

